# Multiomics Analysis Reveals CTHRC1+ CAFs Drive Immunosuppressive Niches and Predict Immunotherapy Resistance in Gastric Cancer

**DOI:** 10.1155/humu/2370955

**Published:** 2026-07-18

**Authors:** Yingxin Wu, Ling-han Tang, Ping Li, Jianwei Xie, Chaohui Zheng, Changming Huang, Hua-ping Ye, Yan-jun Liu

**Affiliations:** ^1^ Section for Gastrointestinal Surgery, Department of General Surgery, The Third People′s Hospital of Chengdu, Chengdu, Sichuan, China; ^2^ Department of General Surgery, Fujian Medical University Union Hospital, Fuzhou, Fujian, China, fjmu.edu.cn; ^3^ Key Laboratory of Ministry of Education of Gastrointestinal Cancer, Fujian Medical University, Fuzhou, Fujian, China, fjmu.edu.cn; ^4^ Department of Gastrointestinal Surgery, Clinical Medical College and The First Affiliated Hospital of Chengdu Medical College, Chengdu, Sichuan, China

**Keywords:** cancer-associated fibroblasts, gastric cancer, immune exclusion, immunotherapy, multiomics

## Abstract

Cancer‐associated fibroblasts (CAFs) orchestrate immune‐excluded tumor microenvironment (TME), but the CAF heterogeneity remains incompletely understood in gastric cancer (GC). In this study, we integrated multicohort single‐cell RNA sequencing (scRNA‐seq), spatial transcriptomics, and bulk transcriptomic data to construct a comprehensive atlas of the GC TME. Unsupervised clustering identified eight transcriptionally distinct CAF subpopulations, among which CTHRC1+ CAFs were selectively enriched in tumors and showed the strongest association with T cell exclusion. Pseudotemporal trajectory analysis, gene regulatory network inference, and cell–cell communication analysis revealed that basic helix‐loop‐helix family member e41 (BHLHE41) serves as a key transcription factor driving CTHRC1+ CAF differentiation, whereas spatial analyses demonstrated these fibroblasts contribute to fibrotic niches at the tumor–stroma interface through macrophage migration inhibitory factor (MIF)–mediated signaling. Finally, we developed and validated a CTHRC1+ cancer‐associated fibroblast–related risk signature (CRS) that accurately predicts immunotherapy response across independent cohorts. These findings establish CTHRC1+ CAFs as a critical stromal determinant of immune exclusion in GC, suggesting that targeting the CTHRC1+ CAF‐MIF axis or applying CRS‐guided patient stratification may enhance immunotherapy efficacy.

## 1. Introduction

Gastric cancer (GC) remains one of the leading causes of cancer‐related mortality worldwide, with limited therapeutic options for advanced‐stage patients [1]. Although immune checkpoint blockade (ICB) has emerged as a promising treatment strategy, its clinical efficacy is substantially hampered by primary and acquired resistance, particularly in tumors exhibiting an immune‐excluded phenotype [2, 3]. In this context, the tumor microenvironment (TME) plays a pivotal role in shaping immune responses, with cancer‐associated fibroblasts (CAFs) representing key stromal architects that establish physical and chemical barriers preventing cytotoxic T cell infiltration [4, 5].

CAFs constitute a remarkably heterogeneous population, encompassing functionally distinct subtypes such as myofibroblastic cancer‐associated fibroblasts (myCAFs), inflammatory cancer‐associated fibroblasts (iCAFs), antigen‐presenting cancer‐associated fibroblasts (apCAFs), and vascular cancer‐associated fibroblasts (vCAFs) [6]. Recent single‐cell RNA sequencing (scRNA‐seq) studies have revealed that specific CAF subsets are selectively enriched in tumors and actively contribute to immune exclusion through extracellular matrix (ECM) remodeling and immunosuppressive signaling [7]. However, the comprehensive landscape of CAF heterogeneity in GC and the precise molecular mechanisms underlying their immunomodulatory functions remain incompletely characterized [6].

Beyond their individual functions, CAFs engage in extensive crosstalk with immune cells, particularly macrophages, to establish self‐reinforcing feedback loops that amplify stromal fibrosis and immune evasion [8]. This CAF‐macrophage axis represents an attractive therapeutic target, yet the specific ligand–receptor interactions and spatial organization of these stromal–immune niches in GC are poorly defined. Furthermore, the lack of robust biomarkers to identify patients who might benefit from stroma‐targeted therapies limits clinical translation.

To address these knowledge gaps, we integrated multicohort scRNA‐seq, spatial transcriptomics, and bulk transcriptomic analyses to construct a comprehensive atlas of the GC TME. We identified eight transcriptionally distinct CAF subpopulations, among which CTHRC1+ CAFs were selectively enriched in tumors and exhibited strong activation of TGF‐*β*, PI3K‐AKT, and ECM remodeling pathways. Through pseudotemporal trajectory analysis and gene regulatory network inference, we uncovered BHLHE41 as a master transcription factor driving CTHRC1+ CAF differentiation. Spatial analyses demonstrated that CTHRC1+ CAFs colocalize with T/NK cells at the tumor–stroma interface, forming fibrotic niches that exclude immune cells via MIF‐mediated signaling. Importantly, we developed a CTHRC1+ cancer‐associated fibroblast–related risk signature (CRS) that accurately predicts immunotherapy response and patient prognosis across independent cohorts. Our findings establish a mechanistic framework for understanding immune exclusion in GC and provide a foundation for developing combination therapeutic strategies targeting the CTHRC1+ CAF to enhance immunotherapy efficacy.

## 2. Methods and Materials

### 2.1. Data Acquisition

#### 2.1.1. scRNA‐seq Data

Four publicly available scRNA‐seq datasets of GC were obtained from the Gene Expression Omnibus (GEO) database: GSE163558, GSE167297, GSE183904, and GSE184198 [9–12]. These datasets encompassed a total of 56 samples (normal and tumor tissues) and 198,199 cells after quality control. Spatial transcriptomics data. Spatial transcriptomics data from four GC tissue sections (GSM7990473, GSM7990474, GSM7990475, and GSM7990476) were retrieved from GSE251950 [13].

#### 2.1.2. Bulk RNA Sequencing Data

One independent bulk RNA‐seq cohort was collected for validation: TCGA‐STAD (The Cancer Genome Atlas Stomach Adenocarcinoma) [14]. Additionally, two immunotherapy‐treated cohorts (PRJEB23709 [15] and GSE91061 [16]) were obtained to evaluate the predictive value of the CRS for immunotherapy response.

### 2.2. Single‐Cell Data Processing and Quality Control

The Seurat package (v4.3.0) in R was applied for downstream analyses. Low‐quality cells with unique molecular identifiers (UMIs) < 500 or mitochondrial gene content > 15*%* were filtered out. Potential doublets were identified and removed using DoubletFinder (v2.0.4). For each dataset, gene expression values were normalized using the LogNormalize method with a scale factor of 10,000. The Top 2000 variable genes were selected using the FindVariableFeatures function (selection.method =  ^“^vst^”^) for subsequent dimensionality reduction.

### 2.3. Data Integration and Batch Correction

Batch effects among samples were corrected using the Harmony integration algorithm [17]. Individual datasets were first normalized and scaled, followed by principal component analysis (PCA) on the Top 2000 variable genes. The first 30 principal components were used as input for Harmony integration with default parameters. Postintegration clustering was performed using FindNeighbors and FindClusters (resolution = 0.8), and results were visualized using Uniform Manifold Approximation and Projection (UMAP) with the first 30 Harmony‐corrected dimensions.

### 2.4. Cell Type Annotation and Subpopulation Identification

Major cell lineages were annotated based on canonical marker expression: B/plasma cells (CD79A, CD79B, MS4A1, and IGKC), T/NK cells (CD3D, CD3E, and NKG7), epithelial cells (EPCAM, KRT18, and KRT19), fibroblasts (COL1A1, COL1A2, and DCN), smooth muscle cells (SMCs; ACTA2 and TAGLN), myeloid cells (CD68, LYZ, and C1QB), endothelial cells (PECAM1 and VWF), and mast cells (CPA3 and TPSAB1). For CAF subtyping, fibroblast populations were extracted and reclustered at higher resolution (resolution = 1.2). Differentially expressed genes (DEGs) were identified using FindAllMarkers with thresholds of min.pct = 0.25, logfc.threshold = 0.25, and only.pos = TRUE. Statistical significance was evaluated using the Wilcoxon rank‐sum test with Benjamini–Hochberg correction for multiple testing.

### 2.5. Functional Annotation of CAF Subtypes

CAF subtypes were functionally annotated using the AUCell package (v1.20.2) to compute enrichment scores for established CAF signatures: myCAF, iCAF, apCAF, and vCAF. Tissue preference was quantified using the Ro/e (ratio of observed to expected cell number) score, where Ro/e > 1 indicates enrichment, 0.8 < Ro/e ≤ 1 indicates slight enrichment, 0.2 ≤ Ro/e ≤ 0.8 indicates depletion, 0 < Ro/e < 0.2 indicates significant depletion, and Ro/e = 0 indicates absence. Pathway activity was inferred using PROGENy (v1.24.0) [18], and KEGG pathway enrichment analysis was conducted using the clusterProfiler package (v4.6.2) [19] with terms considered significant at adjusted *p* < 0.05.

### 2.6. Pseudotemporal Trajectory Analysis

CAF differentiation trajectories were reconstructed using Monocle 2 (v2.26.0) [20]. Prior to trajectory analysis, the fibroblast subset was batch‐corrected using Harmony, and cell clusters were identified based on the Harmony reduction with FindNeighbors (dims = 1 : 20) and FindClusters (resolution = 0.3). The Top 100 DEG distinguishing CAF subclusters were used to define the trajectory. Dimensionality reduction was performed using DDRTree (max_components = 2), pseudotime‐associated genes were identified by differentialGeneTest, and branch‐dependent programs were analyzed using BEAM (branched expression analysis modeling) and visualized with plot_genes_branched_pseudotime.

### 2.7. Transcription Factor Regulon Inference

Transcriptional regulatory networks were reconstructed using pySCENIC (v0.12.0) [21], which calculates regulon activity based on coexpression and motif enrichment. The analysis pipeline comprised three steps: (1) identification of coexpression modules using GRNBoost2, (2) generation of regulons via cisregulatory motif enrichment analysis using cisTarget, and (3) quantification of regulon activity using AUCell. Subtype‐specific transcription factors were identified by the Wilcoxon test, and regulon specificity scores (RSS) were computed using Jensen–Shannon divergence via the philentropy package (v0.6.0).

### 2.8. High‐Dimensional Weighted Gene Coexpression Network Analysis (hdWGCNA)

hdWGCNA was performed to identify transcriptional modules associated with specific CAF subtypes [22]. Scale‐free topology analysis was conducted to determine the optimal soft‐thresholding power (power = 12, *R* ^2^ > 0.9). The topological overlap matrix (TOM) was calculated, and hierarchical clustering was performed to identify gene modules (minimum module size = 30). Module eigengenes were computed, and module‐trait correlations were assessed. Hub genes were defined as those with high module membership (kME > 0.8) and intramodular connectivity.

### 2.9. Cell–Cell Communication Analysis

Potential interactions between cell types were predicted using CellChat (v2.2.0) with CellChatDB.human as the reference database [23]. A significance threshold with *p* value < 0.05 was used to predict cell–cell interactions between different cell types. To further characterize crosstalk between CTHRC1+ CAFs and immune cells, the NicheNet package was employed to infer ligand–target interactions. The Top 20 ligands and 100 downstream targets were used to compute ligand‐target activity (score range: 0–1). Heatmaps displaying average expression across subtypes were generated after scaling.

### 2.10. Spatial Transcriptomics Analysis

Spatial gene‐spot matrices from Visium data were analyzed using Seurat (v4.3.0). Spots expressing fewer than 200 genes or genes detected in fewer than three spots were filtered out, and data were normalized using SCTransform. PCA was performed using the first 30 principal components. Single‐cell‐derived signature scores were mapped to spatial data using AddModuleScore and visualized with SpatialFeaturePlot. Cell type deconvolution was performed using RCTD (Robust Cell Type Decomposition). Spatial colocalization analysis was conducted using heatmaps to quantify spatial correlation between different populations, and pathway dependency analysis was performed to assess local microenvironmental signaling.

### 2.11. Construction and Validation of CRS

The CRS was constructed based on overlapping genes between hdWGCNA‐derived module genes (M1, M2, M5, and M10) and CTHRC1+ CAF marker genes. Risk scores were calculated for each patient using the ssGSEA algorithm. Patients were stratified into high‐ and low‐CRS groups using the median risk score as cutoff. The predictive performance was evaluated in two independent immunotherapy cohorts (PRJEB23709 and GSE91061) using receiver operating characteristic (ROC) curves, Kaplan–Meier survival analysis, and response rate comparison.

### 2.12. TME and Genomic Analysis

ESTIMATE algorithm was used to calculate stromal scores, immune scores, and tumor purity [24]. CIBERSORT was employed to infer relative proportions of 22 immune cell types [25]. Tumor mutational burden (TMB) was calculated as the total number of nonsynonymous mutations per megabase. Immunophenoscore (IPS) was obtained from The Cancer Immunome Atlas (TCIA) [26]. Genomic alteration analysis including copy number variations (CNVs) was performed using GISTIC2.0 outputs from TCGA.

### 2.13. Drug Sensitivity Analysis

Drug sensitivity profiling was performed using the CTRP [27] and PRISM databases [28]. Estimated half‐maximal inhibitory concentration (IC50) values were calculated using the oncoPredict package. Correlation between CRS and drug response was assessed using Pearson correlation, and differential sensitivity between risk groups was evaluated using the Wilcoxon rank‐sum test.

### 2.14. Statistical Analysis

All statistical analyses were performed in R (v4.3.1). Continuous variables were compared using Wilcoxon rank‐sum test or Student′s *t*‐test as appropriate. Categorical variables were analyzed using chi‐square test or Fisher′s exact test. Survival analysis was conducted using Kaplan–Meier method with log‐rank test. Correlations were assessed using Pearson or Spearman correlation coefficients. Multiple testing correction was performed using Benjamini–Hochberg method where applicable. A *p* value < 0.05 was considered statistically significant.

## 3. Results

### 3.1. Dissecting the TME and Heterogeneity of CAFs in the GC

**Figure 1:**
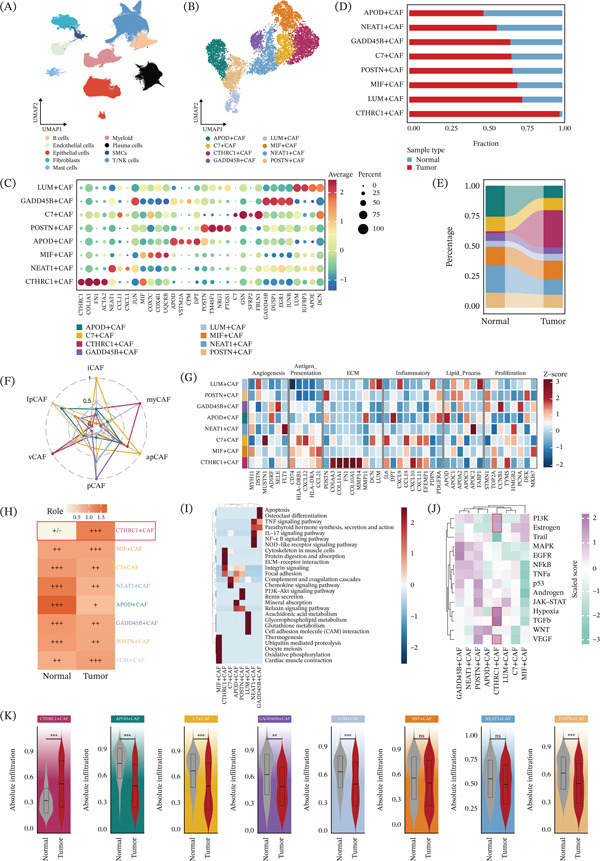
Heterogeneity and functional annotation of cancer‐associated fibroblast (CAF) subpopulations in gastric cancer. (A) Nine major cell types: B cells, plasma cells, T/NK cells, epithelial cells, fibroblasts, smooth muscle cells (SMCs), myeloid cells, endothelial cells, and mast cells are annotated in the gastric cancer tumor microenvironment. (B) UMAP plot of reclustered fibroblast populations reveals eight transcriptionally distinct CAF subpopulations. (C) Dot plot shows the Top 4 differentially expressed marker genes for each CAF subtype. (D) Bar plot compares the proportion of each CAF subtype in normal versus tumor tissues. CTHRC1+, MIF+, and POSTN+ CAFs are significantly enriched in tumors. (E) Sankey diagram illustrates the proportional shift of CAF composition from normal to tumor microenvironments. (F) Radar plot depicts the enrichment scores of established CAF signatures (myCAF, iCAF, apCAF, and vCAF) across the eight subtypes. (G) A heatmap shows the expression of signature gene sets corresponding to different CAF functional categories. (H) Ro/e (ratio of observed to expected) scores quantifies the tissue preference of each CAF subtype. The number of “+” signs indicates the strength of tissue preference based on the Ro/e score, defined as follows: Ro/e > 1 indicates enrichment, 0.8 < Ro/e ≤ 1 indicates slight enrichment, 0.2 ≤ Ro/e ≤ 0.8 indicates depletion, 0 < Ro/e < 0.2 indicates significant depletion, and Ro/e = 0 indicates absence. (I) KEGG pathway enrichment analysis for subtype‐specific differentially expressed genes. (J) Heatmap of pathway activity scores are inferred by PROGENy across CAF subtypes. (K) CAF subtype abundance in tumors compared with normal tissues of the TCGA‐STAD cohort. ns indicates not significant;  ^∗∗^ indicates *p* < 0.01;  ^∗∗∗^ indicates *p* < 0.001.

Four STAD scRNA‐seq datasets (GSE163558, GSE167297, GSE183904, and GSE184198) were integrated. A total of 198,199 cells were included, and 13 distinct cell clusters were identified (Figure S1A), annotated into nine major cell types including immune, stromal, and epithelial populations (Figure S1B). Although GSE183904 contributed most cells (74.58%) and samples (64.29%), all datasets were proportionally represented across cell types (Figure S1C–E). Consistent sequencing depths across populations (Figure S1E, right) confirmed successful batch correction and data reliability for subsequent analyses. As shown in Figure 1A, cells were clustered into nine major cell types: B cells, plasma cells, T/NK cells, epithelial cells, fibroblasts, SMCs, myeloid cells, endothelial cells, and mast cells. To dissect the cellular heterogeneity of CAFs in GC, we performed unsupervised clustering analysis of fibroblast populations, which revealed eight transcriptionally distinct CAF subpopulations (Figures S2A and 1B): APOC1+ CAF, C7+ CAF, CTHRC1+ CAF, GADD45B+ CAF, LUM+ CAF, MIF+ CAF, NEAT1+ CAF, and POSTN+ CAF. Each subtype was characterized by unique marker gene signatures, as visualized by dot plot analysis showing the Top 4 DEGs per subpopulation (Figure 1C). UMAP visualization by tissue origin revealed marked differences in subpopulation distribution between normal and tumor samples, with specific subsets selectively enriched in malignant tissues (Figure S2B). Cell cycle analysis demonstrated heterogeneous proliferative states across CAF subtypes, with CTHRC1+ CAF and POSTN+ CAF exhibiting higher proportions of cycling cells (Figure S2C). Functional annotation using established CAF signatures showed distinct enrichment patterns: CTHRC1+ CAF and POSTN+ CAF displayed prominent myCAF scores, GADD45B+ CAF and NEAT1+ CAF showed iCAF characteristics, and APOC1+ CAF exhibited apCAF features, whereas C7+ CAF demonstrated vCAF properties (Figure S2D). Transcriptomic analysis revealed specialized ECM remodeling programs across CAF subtypes. CTHRC1+ CAF and POSTN+ CAF highly expressed collagen genes (COL1A1, COL1A2, and COL3A1) and matrix metalloproteinases (MMP11 and MMP14), consistent with their myofibroblastic, matrix‐depositing phenotype (Figure S2E–F). In contrast, GADD45B+ CAF and NEAT1+ CAF showed elevated expression of lysyl oxidases (LOX and LOXL2) involved in collagen cross‐linking and tissue stiffness. Tissue preference quantification using odds ratio (OR) scores confirmed significant enrichment of CTHRC1+ CAF, POSTN+ CAF, and MIF+ CAF in tumor tissues (Figure S2G). In addition, the CTHRC1+ CAF subtype demonstrates the highest tumor‐associated distribution patterns (Figure 1D). Sankey plot further illustrated the proportional shift of CAF composition between normal and TMEs (Figure 1E). To functionally annotate these subpopulations, we employed AUCell algorithm‐based scoring of established CAF signatures. Radar plot analysis revealed that CTHRC1+ CAF and POSTN+ CAF exhibited prominent myCAF characteristics, characterized by high expression of ECM remodeling genes; GADD45B+ CAF and NEAT1+ CAF displayed iCAF features with elevated cytokine and chemokine signaling; APOC1+ CAF showed apCAF potential with enhanced MHC Class II expression; and C7+ CAF demonstrated vCAF properties associated with angiogenic factors (Figure 1F). These functional assignments were corroborated by heatmap analysis of relevant gene signatures across all eight subtypes (Figure 1G). Tissue preference quantification using Ro/e scores confirmed the significant enrichment of myCAF‐like subtypes (CTHRC1+ CAF, MIF+ CAF, POSTN+ CAF) in tumor tissues, whereas LUM+ CAF was exclusively associated with normal gastric mucosa (Figure 1H). KEGG pathway enrichment analysis of subtype‐specific DEGs revealed distinct functional specializations: ECM‐receptor interaction and focal adhesion pathways were prominently activated in POSTN+ CAF and CTHRC1+ CAF, cytokine–cytokine receptor interaction and NF‐*κ*B signaling were enriched in GADD45B+ CAF, antigen processing and presentation characterized APOC1+ CAF, and lipid metabolism pathways were notable in C7+ CAF (Figure 1I). PROGENy pathway activity inference demonstrated differential oncogenic signaling across CAF subtypes, with tumor‐enriched populations (CTHRC1+ CAF, POSTN+ CAF, and MIF+ CAF) showing enhanced activation of PI3K, MAPK, TGF*β*, and hypoxia pathways compared with normal tissue‐associated LUM+ CAF (Figure 1J). Finally, we validated the clinical relevance of these CAF subtypes by analyzing their absolute infiltration levels in the TCGA‐STAD cohort. Consistent with our scRNA‐seq findings, CTHRC1+ CAF, POSTN+ CAF, and MIF+ CAF demonstrated significantly elevated abundance in gastric tumors compared with adjacent normal tissues (Figure 1K). Because CTHRC1+ CAF is most significantly elevated in cancer tissues compared with normal, we chose CTHRC1+ CAF for further investigation.

### 3.2. Pseudotemporal Analysis Reveals Developmental Trajectories and Regulatory Networks of CAF Subtypes in GC

To investigate the developmental relationships and dynamic transitions among CAF subpopulations, we performed pseudotime analysis using Monocle 2. The reconstructed trajectory revealed a bifurcated differentiation path originating from LUM+ CAF and progressing toward two distinct terminal states predominantly occupied by CTHRC1+ CAF/POSTN+ CAF and MIF+ CAF, respectively (Figure S3A). Pseudotime values, ranging from 0 (early) to 12 (late), demonstrated progressive cellular maturation along this trajectory (Figure S3B). State partitioning identified nine distinct cellular states, with proportional representation analysis revealing that LUM+ CAF primarily occupied early states (States 1–3), whereas myofibroblast‐like subtypes (CTHRC1+ CAF, POSTN+ CAF, and MIF+ CAF) were enriched in terminal states (States 7–9), suggesting a potential differentiation cascade from normal tissue‐associated fibroblasts toward tumor‐promoting CAF phenotypes (Figure S3C). BEAM identified four major gene clusters with distinct temporal expression patterns. Cluster 1 genes, including LUM and DCN, exhibited high early expression that decreased along pseudotime, consistent with quiescent fibroblast signatures. Cluster 2 and 3 genes showed transient upregulation at intermediate pseudotime values, representing transitional activation states. Notably, Cluster 4 genes, encompassing ECM remodeling factors (POSTN and MMP11) and hypoxia‐response genes (VEGFA and SLC2A1), demonstrated progressive upregulation toward late pseudotime, coinciding with myCAF terminal differentiation (Figure S3D,E). GO analysis confirmed that early states were enriched for ECM organization and cell adhesion, whereas late states were characterized by hypoxia response, glycolytic process, and positive regulation of cell migration (Figure S3D, right). To elucidate the transcriptional regulatory mechanisms governing CAF heterogeneity, we inferred gene regulatory networks using pySCENIC. RSS ranking identified the Top 6 specific master regulators for CTHRC1+ CAF subpopulation (Figure S3F). Strikingly, BHLHE41 emerges as the most specifically activated TF in CTHRC1+ CAFs, with both its mRNA expression (Figure S3G) and regulon activity (Figure S3H) showing exclusive enrichment in this subtype. UMAP visualization and violin plots confirmed minimal BHLHE41 expression in other CAF populations, suggesting its potential role as a master regulator driving the myofibroblastic and tumor‐promoting phenotype of CTHRC1+ CAF.

### 3.3. CTHRC1+ CAFs Are Associated With T Cell Exclusion in GC

**Figure 2:**
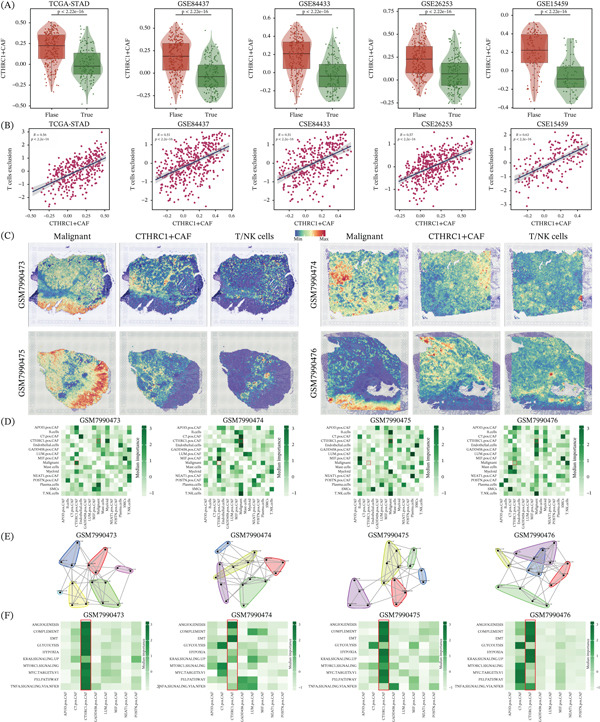
CTHRC1+ CAFs are spatially associated with T cell exclusion. (A) Box‐violin plots comparing CTHRC1+ CAF signature scores between responders (R) and nonresponders (NR) to immunotherapy across five independent gastric cancer cohorts. (B) Correlation heatmap showing Pearson correlation coefficients between CAF subtype signature scores and T cell exclusion scores across the same cohorts. (C) Spatial transcriptomics analysis: Spatial feature plots show the distribution of malignant cells, CTHRC1+ CAFs, and T/NK cells as deconvoluted by RCTD. (D) Heatmap of spatial colocalization scores (correlation) between CTHRC1+ CAFs, malignant cells, and T/NK cells across the four spatial samples. (E) Network diagram visualizing cell–cell interaction dependencies, with CTHRC1+ CAFs as a central node connecting to malignant cells and T/NK cells. (F) Heatmap of pathway dependency scores in spatial spots enriched for CTHRC1+ CAFs, highlighting associated biological processes.

To investigate the clinical relevance of CTHRC1+ CAFs, we first examined their association with immunotherapy response across five independent bulk RNA‐seq cohorts (TCGA‐STAD, GSE84437, GSE84433, GSE26253, and GSE15459). Strikingly, CTHRC1+ CAF signature scores were significantly elevated in nonresponders (NR) compared with responders in all cohorts (all *p* < 2.2 × 10^−16^), suggesting that high CTHRC1+ CAF abundance predicts poor immunotherapy outcome (Figure 2A). Given the established role of CAFs in shaping immune cell infiltration, we next assessed the relationship between CAF subtype signatures and T cell exclusion scores. Pearson correlation analysis revealed that CTHRC1+ CAF showed the strongest positive correlation with T cell exclusion across all five cohorts (*R* = 0.58–0.63, all *p* < 2.2 × 10^−16^), whereas other CAF subtypes exhibited weaker or inconsistent associations (Figure 2B). These findings implicate CTHRC1+ CAFs as a key stromal mediator of T cell exclusion in GC. To validate these observations at spatial resolution, we analyzed four GC spatial transcriptomics samples (GSM7990473–0476) using RCTD deconvolution. Spatial feature plots demonstrated distinct distribution patterns: Malignant cells exhibited clustered localization in tumor nests, CTHRC1+ CAFs were enriched at the tumor–stroma interface, and T/NK cells were predominantly excluded from tumor cores and confined to peripheral stromal regions (Figure 2C). Notably, samples with higher CTHRC1+ CAF infiltration (GSM7990473 and GSM7990475) showed more pronounced T/NK cell exclusion compared with those with lower CTHRC1+ CAF abundance (GSM7990474 and GSM7990476). Cell type colocalization analysis using heatmaps revealed significant positive spatial correlation between CTHRC1+ CAFs and malignant cells, and negative correlation between CTHRC1+ CAFs and T/NK cells across all four sections (Figure 2D). Network visualization of cell–cell interaction dependencies further demonstrated that CTHRC1+ CAFs occupied central nodes in the stromal–immune interaction network, with strong reciprocal connections to malignant cells and inhibitory edges to T/NK cell populations (Figure 2E). Pathway dependency analysis indicated that CTHRC1+ CAF‐mediated T cell exclusion was associated with enhanced ECM remodeling, TGF‐*β* signaling, and immune checkpoint molecule expression in the local microenvironment (Figure 2F). Furthermore, spatial transcriptomic analysis of four GC samples (GSM7990473–GSM7990476) using RCTD deconvolution revealed distinct cell type distributions across tissue sections (Figure S4A). CTHRC1+ CAFs exhibited specific localization patterns, with homologous cell network analysis demonstrating their spatial clustering and heterotypic interactions with malignant cells (Figure S4B,C). Notably, CTHRC1+ CAFs showed preferential colocalization with tumor cells at the invasive front, forming a dense stromal barrier adjacent to cancer nests. Analysis of immune checkpoint molecule expression in the TCGA‐STAD cohort revealed significantly elevated levels of multiple inhibitory receptors in CTHRC1+ CAF‐high tumors, including CD274 (PD‐L1), PDCD1 (PD‐1), LAG3, TIGIT, HAVCR2 (TIM‐3), PDCD1LG2 (PD‐L2), CD276 (B7‐H3), ICOS, CD80, CD86, TNFRSF9 (4‐1BB), and TNFRSF4 (OX40) (all *p* < 0.05) (Figure S4D). This immunosuppressive molecular profile suggests that CTHRC1+ CAFs contribute to an immune‐inhibitory microenvironment beyond physical barrier formation. GSVA analysis of Hallmark pathway activities across CAF subtypes demonstrated distinct functional specialization (Figure S4E). CTHRC1+ CAFs exhibited prominent activation of epithelial‐mesenchymal transition (EMT), TGF‐*β* signaling, hypoxia, and glycolysis pathways, consistent with their myofibroblastic and metabolically reprogrammed phenotype. In contrast, other subtypes showed differential enrichment: GADD45B+ CAF and NEAT1+ CAF in inflammatory response and interferon‐*γ* signaling; APOC1+ CAF in allograft rejection and complement activation, whereas LUM+ CAF displayed relatively quiescent pathway activities.

### 3.4. hdWGCNA Reveals Transcriptional Modules Associated With Distinct CAF Subpopulations

**Figure 3:**
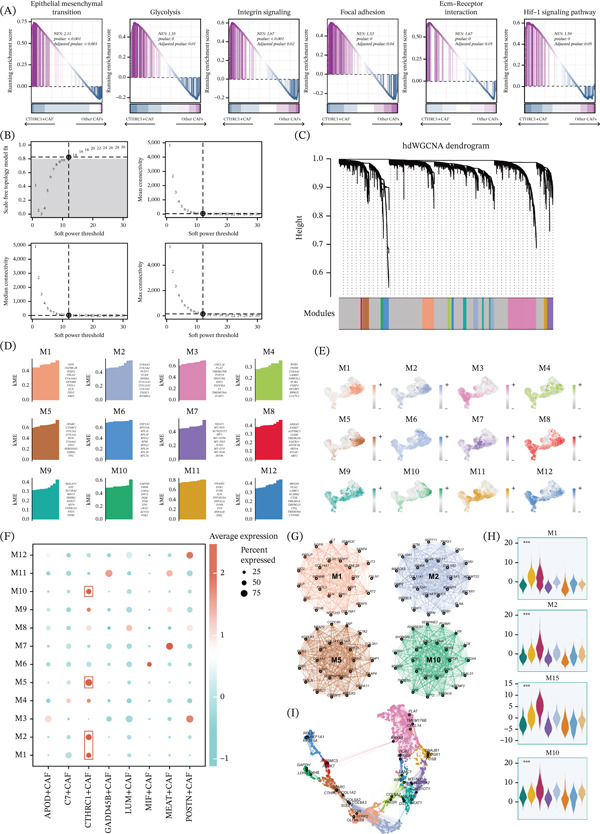
hdWGCNA identifies coexpression modules specific to CTHRC1+ CAFs. (A) GSEA enrichment plots showing hallmark pathways upregulated in CTHRC1+ CAFs compared with other subtypes. (B) Scale‐free topology fit index and mean connectivity analysis for selecting the soft‐thresholding power (*β* = 12) in hdWGCNA. (C) Hierarchical clustering dendrogram of genes and module color assignment, identifying 12 distinct coexpression modules (M1–M12). (D) The Top 10 hub genes for each of the 12 modules. (E) UMAP visualization of CAF cells, colored by the eigengene score of four selected CTHRC1+ CAF‐enriched modules (M1, M2, M5, and M10). (F) Dot plot showing the average module eigengene expression across all CAF subtypes, confirming enrichment of M1, M2, M5, and M10 in CTHRC1+ CAFs. (G) Network visualization of intramodular connectivity among hub genes within the four CTHRC1+ CAF‐enriched modules. (H) Violin plots compare the eigengene scores of modules M1, M2, M5, and M10 between CTHRC1+ CAFs and all other subtypes combined.  ^∗∗∗^ indicates *p* < 0.001. (I) Integrated network graph displays all hub genes from the 12 modules, with coherent coexpression patterns highlighted within the CTHRC1+ CAF population.

To elucidate the molecular programs underlying CTHRC1+ CAF‐mediated tumor promotion, we first performed GSEA comparing CTHRC1+ CAFs with all other CAF subtypes. CTHRC1+ CAFs showed significant enrichment for EMT, glycolysis, integrin signaling, focal adhesion, ECM‐receptor interaction, and TGF‐*β* signaling pathways (all NES > 1.5, FDR < 0.05), indicating their activated myofibroblastic and metabolic reprogramming phenotype (Figure 3A). To identify coregulated gene networks driving CTHRC1+ CAF identity, we applied hdWGCNA. Scale‐free topology analysis identified soft − threshold power = 12 as the optimal parameter for network construction (Figure 3B). Hierarchical clustering of the TOM revealed 12 distinct transcriptional modules (M1–M12) with unique color assignments (Figure 3C). Each module was characterized by its Top 10 hub genes, which exhibited high module membership scores and intramodular connectivity (Figure 3D). UMAP visualization of module eigengene scores across all CAF subpopulations revealed distinct spatial distribution patterns, with specific modules showing restricted enrichment in particular subtypes (Figure 3E). Dot plot analysis demonstrated that modules M1, M2, M5, and M10 were preferentially activated in CTHRC1+ CAFs compared with other subpopulations, including APOC1+ CAF, C7+ CAF, GADD45B+ CAF, LUM+ CAF, MIF+ CAF, NEAT1+ CAF, and POSTN+ CAF (Figure 3F). Network analysis of these CTHRC1+ CAF‐enriched modules identified robust hub gene interactions within M1 (cell cycle and proliferation), M2 (ECM organization and collagen fibril assembly), M5 (TGF‐*β* signaling and myofibroblast differentiation), and M10 (hypoxia response and angiogenesis) (Figure 3G). Violin plots confirmed significantly elevated module eigengene scores for M1, M2, M5, and M10 in CTHRC1+ CAFs compared with all other subtypes (all *p* < 0.001), establishing these transcriptional programs as CTHRC1+ CAF‐specific signatures (Figure 3H). Finally, integrated visualization of all 12 module hub gene networks demonstrated coherent coexpression patterns specifically within the CTHRC1+ CAF population, reinforcing the modular and hierarchical organization of their tumor‐promoting transcriptional program (Figure 3I).

### 3.5. CTHRC1+ CAFs May Inhibit T Cells Activity Through MIF Signaling Pathway

**Figure 4:**
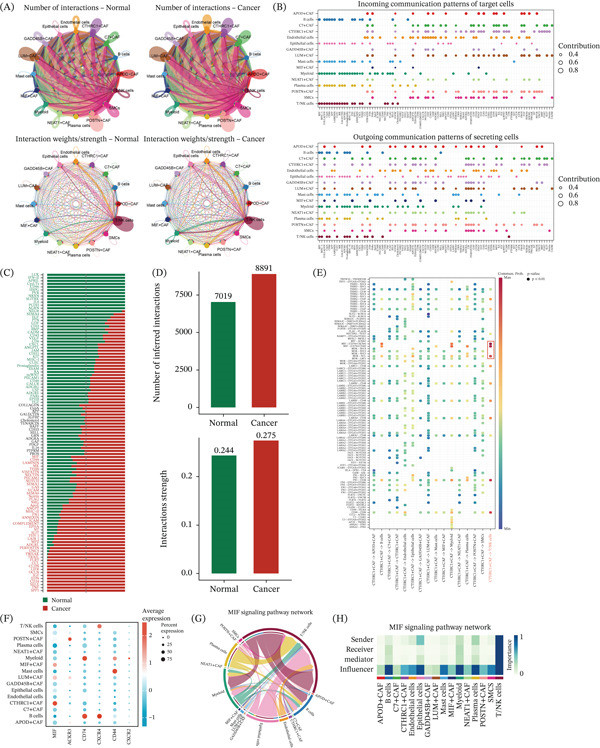
Cell–cell communication analysis implicates MIF signaling may serve as a key immunosuppressive regulator from CTHRC1+ CAFs. (A) Circle plots comparing the number and strength of inferred cell–cell interactions from CTHRC1+ CAFs in normal versus tumor tissues. (B) Heatmap showing the communication patterns of CTHRC1+ CAFs as senders or receivers of signals across major cell types. (C) Bar plot comparing the activity of selected signaling pathways between tumor and normal tissues. (D) Bar charts quantifying the total number of interactions and overall interaction strength in tumor versus normal microenvironments. (E) Bubble plot displaying the expression of key ligand–receptor pairs involved in CTHRC1+ CAF‐specific communication, with MIF signaling highlighted. (F) Dot plot shows expression of the MIF ligand in CTHRC1+ CAFs and its receptors (CD74, CXCR4, and CD44) in T/NK cells and myeloid cells. (G) Chord diagram illustrating the extensive MIF signaling network originating from CTHRC1+ CAFs to various receiver cell types. (H) Network centrality analysis identifying CTHRC1+ CAFs as the dominant sender within the MIF signaling network.

To investigate how CTHRC1+ CAFs mediate immune suppression, we performed cell–cell communication analysis using CellChat. Circular network plots revealed substantially enhanced interaction numbers and weights between CTHRC1+ CAFs and other cell populations in cancer compared with normal tissues, with CTHRC1+ CAFs serving as prominent signaling hubs in the TME (Figure 4A). Communication pattern analysis demonstrated that CTHRC1+ CAFs primarily acted as signal senders through autocrine and paracrine mechanisms, whereas T/NK cells and myeloid cells were predominant receivers of CTHRC1+ CAF‐derived signals (Figure 4B). Comparative analysis of signaling pathway activities between tumor and adjacent normal tissues identified multiple pathways with significantly elevated interaction strength in cancer, including MIF, CXCL, COMPLEMENT, and TGF‐*β* (Figure 4C). Quantification confirmed that cancer tissues exhibited both increased total interaction numbers (8891 vs. 7019) and enhanced overall interaction strength (0.275 vs. 0.244) compared with normal tissues (Figure 4D). Focused analysis of CTHRC1+ CAF‐specific ligand–receptor pairs revealed that MIF signaling represented the most prominent communication axis between CTHRC1+ CAFs and immune cells (Figure 4E). Expression analysis showed that CTHRC1+ CAFs highly expressed MIF ligand, whereas cognate receptors CD74, CXCR4, and CD44 were predominantly expressed on T/NK cells, macrophages, and dendritic cells (Figure 4F). Chord diagram visualization illustrated extensive MIF‐mediated connections originating from CTHRC1+ CAFs and targeting diverse immune and stromal cell types within the tumor ecosystem (Figure 4G). Network centrality analysis further established CTHRC1+ CAFs as the dominant sender and influencer within the MIF signaling network, with T/NK cells and myeloid cells serving as major receivers (Figure 4H).

### 3.6. CRS Can Predict Immunotherapy Response

**Figure 5:**
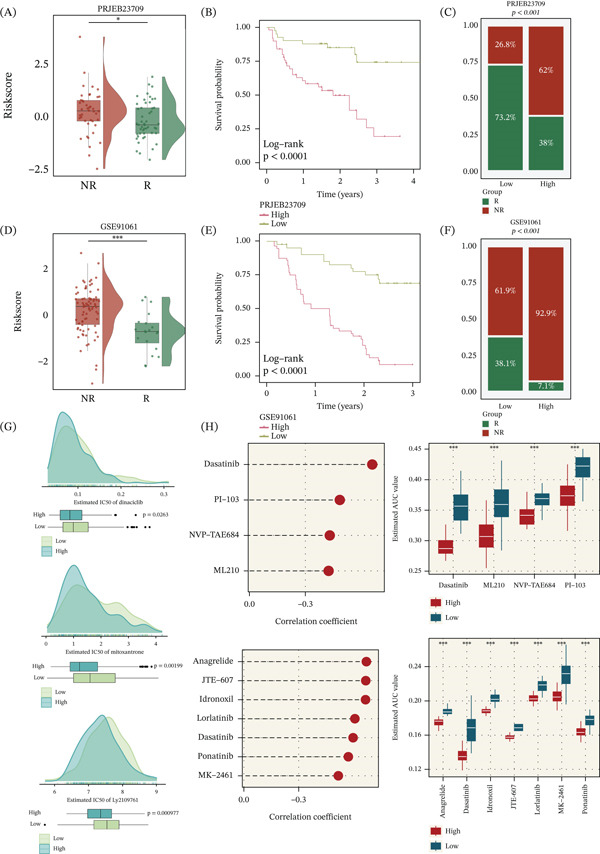
The CTHRC1+ CAF‐related risk signature (CRS) can predict immunotherapy response. (A, D) Box‐violin plots show significantly higher CRS in nonresponders (NR) compared with responders (R) in (A) the PRJEB23709 and (D) GSE91061 immunotherapy cohorts. (B, E) Kaplan–Meier survival curves demonstrating significantly worse overall survival for patients with high CRS in (B) the PRJEB23709 and (E) GSE91061 cohorts. (C, F) Bar charts show the objective response rates in high‐ versus low‐CRS groups in (C) the PRJEB23709 and (F) GSE91061 cohorts. (G) Density distribution and box plots of estimated IC50 values for candidate drugs (dasatinib, mitoxantrone, and LY2109761) showing increased sensitivity in high‐CRS tumors. (H) Scatter plots show significant negative correlation between CRS and drug sensitivity (IC50) for selected compounds; box plots showing differential drug sensitivity between high‐ and low‐CRS groups for additional candidate agents.  ^∗^ indicates *p* < 0.05, and  ^∗∗∗^ indicates *p* < 0.001.

To develop a clinically applicable biomarker, we constructed a CRS based on the overlapping genes from hdWGCNA‐derived module genes and CTHRC1+ CAF marker genes (Figure S4F). In the PRJEB23709 immunotherapy cohort, CRS was significantly elevated in NR compared with responders (R) (*p* < 0.001), demonstrating its predictive value for treatment outcome (Figure 5A). Kaplan–Meier survival analysis revealed that patients with high CRS exhibited significantly worse overall survival compared with those with low CRS (log‐rank *p* < 0.0001) (Figure 5B). Consistently, response rate analysis showed that the low‐CRS group achieved a 73.2% response rate versus only 38% in the high‐CRS group (Figure 5C). We validated these findings in the independent GSE91061 immunotherapy cohort. Similar to the discovery cohort, CRS was markedly higher in NR (Figure 5D), and high‐CRS patients demonstrated significantly inferior survival outcomes (log‐rank *p* < 0.0001) (Figure 5E). The predictive performance was further corroborated by response stratification, with the low‐CRS group showing a 38.1% response rate compared with merely 7.1% in the high‐CRS group (Figure 5F). These results establish CRS as a robust prognostic indicator for immunotherapy efficacy across independent cohorts. Given the therapeutic potential of targeting CTHRC1+ CAF‐associated pathways, we performed drug sensitivity profiling using CTRP and PRISM databases. Density distribution and boxplot analyses revealed that high‐CRS tumors exhibited significantly lower estimated IC50 values for dasatinib, mitoxantrone, and LY2109761, indicating enhanced sensitivity to these agents (Figure 5G). Correlation analysis further identified dasatinib, PI‐103, NVP‐TAE684, and ML210 as top candidate compounds with strong negative correlation between CRS and drug response (Figure 5H, top). Additionally, anagrelide, JTE‐607, idronoxil, lorlatinib, ponatinib, and MK‐2461 showed significant differential sensitivity between high‐ and low‐CRS groups, with high‐CRS tumors being more responsive to these targeted therapies (Figure 5H, bottom).

### 3.7. Comprehensive Analysis of TME, Genomic Characteristics, and Clinical Prognosis Based on CRS Stratification

**Figure 6:**
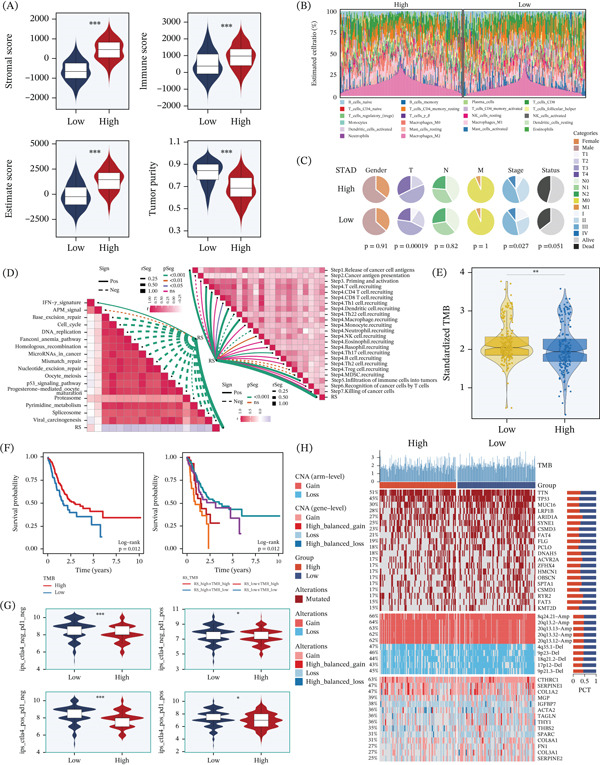
Comprehensive characterization of the tumor microenvironment and genomic landscape stratified by CRS. (A) Box‐violin plots compare ESTIMATE‐derived stromal score, immune score, ESTIMATE score, and tumor purity between high‐ and low‐CRS groups in TCGA‐STAD. (B) Stacked plot shows the relative proportions of 22 immune cell types (CIBERSORT) in high‐ versus low‐CRS tumors. (C) Pie charts of clinical feature associations with CRS status (high/low), including T stage, N stage, M stage, stage, survival status, and gender. (D) Correlation matrix between CRS and various immune signatures (e.g., cytolytic activity, IFN‐*γ* response, and TGF‐*β* signaling). (E) Comparison of tumor mutational burden (TMB) between high‐ and low‐CRS groups. (F) Kaplan–Meier survival analysis combines CRS and TMB, and identifies four distinct prognostic subgroups. (G) Box plots comparing immunophenoscore (IPS) for two treatment scenarios (CTLA4−/PD1− and CTLA4+/PD1+) between CRS groups. (H) Oncoplot shows the landscape of somatic copy number alterations (amplifications in red and deletions in blue) in high‐ versus low‐CRS tumors, focusing on stromal and immune‐related genomic regions.  ^∗^ indicates *p* < 0.05,  ^∗∗^ indicates *p* < 0.01, and  ^∗∗∗^ indicates *p* < 0.001.

To elucidate the biological basis underlying CRS‐mediated immunotherapy resistance, we systematically characterized the TME and genomic landscape of high‐ and low‐CRS patients in TCGA‐STAD cohort. ESTIMATE analysis revealed that high‐CRS tumors exhibited significantly elevated stromal scores, immune scores, and ESTIMATE scores, coupled with reduced tumor purity, indicating an immunosuppressive and stroma‐rich microenvironment (Figure 6A). CIBERSORT deconvolution further demonstrated distinct immune cell composition between risk groups, with high‐CRS tumors showing increased infiltration of immunosuppressive populations including M2 macrophages and regulatory T cells, and decreased cytotoxic lymphocytes (Figure 6B). Clinical correlation analysis showed that high‐CRS status was significantly associated with advanced T stage (*p* = 0.00019) and higher stage (*p* = 0.027), as well as poorer survival status (*p* = 0.051), whereas no significant associations were observed with gender, N stage, or M stage (Figure 6C). Correlation matrix analysis revealed that CRS was negatively associated with multiple ICB response signatures, including cytolytic activity, IFN‐*γ* response, and T cell‐inflamed gene expression profile, and positively correlated with immunosuppressive pathways such as TGF‐*β* signaling and angiogenesis (Figure 6D). TMB analysis demonstrated significantly lower TMB in high‐CRS compared with low‐CRS patients (*p* < 0.01) (Figure 6E). Survival analysis showed that although high TMB alone did not stratify prognosis, the combination of CRS and TMB identified distinct risk groups, with high‐CRS/low‐TMB patients exhibiting the worst outcomes and low‐CRS/high‐TMB patients showing the best survival (*p* < 0.0001) (Figure 6F). IPS analysis through TCIA further confirmed that high‐CRS tumors had significantly lower IPS‐CTLA4_neg_PD1_neg and IPS‐CTLA4_pos_PD1_pos scores, indicating diminished immunogenicity and impaired response to checkpoint inhibition (Figure 6G). Finally, genomic alteration analysis revealed distinct CNV patterns between risk groups, with high‐CRS tumors enriched for amplifications in stromal‐related genes (e.g., COL1A1 and COL3A1) and deletions in tumor suppressor genes, whereas low‐CRS tumors showed higher frequencies of immune‐related gene alterations (Figure 6H).

In addition, we first validated the stromal basis of the CRS by examining its correlation with fibroblast abundance. CRS demonstrated strong positive correlation with fibroblast scores derived from MCPcounter (*R* = 0.967, *p* < 2.2 × 10^−16^), confirming that high‐CRS tumors are enriched in CAF content (Figure S5A). Systematic characterization of immune cell infiltration using ssGSEA revealed distinct immune microenvironment compositions between risk groups. High‐CRS tumors exhibited significantly elevated infiltration of immunosuppressive populations including regulatory T cells, M2 macrophages, and myeloid‐derived suppressor cells, while showing reduced abundance of cytotoxic lymphocytes, natural killer cells, and activated dendritic cells (Figure S5B). Radar plot visualization of immune pathway activities demonstrated that high‐CRS tumors were characterized by enhanced APC coinhibition, T cell coinhibition, and parainflammation signatures, alongside diminished cytolytic activity, Type I IFN response, and T cell costimulation (Figure S5C). Analysis of immune checkpoint molecule expression confirmed the immunosuppressive phenotype of high‐CRS tumors, with significantly elevated expression of CD274 (PD‐L1), PDCD1 (PD‐1), CTLA4, LAG3, TIGIT, HAVCR2 (TIM‐3), PDCD1LG2 (PD‐L2), and CD276 (B7‐H3) (Figure S5D). Evaluation of cancer‐immunity cycle step activities revealed that high‐CRS tumors exhibited impaired function across multiple stages, particularly in T cell priming and activation, trafficking to tumors, and recognition of cancer cells (Figure S5E). Genomic alteration analysis using GISTIC 2.0 identified distinct CNV patterns between risk groups. High‐CRS tumors displayed widespread chromosomal instability with frequent amplifications in regions harboring stromal‐related genes (e.g., 1q21‐23, 8q24) and deletions in tumor suppressor loci (e.g., 9p21 and 18q21) (Figure S5F). Quantification confirmed significantly higher rates of both focal and arm‐level copy number alterations in high‐CRS compared with low‐CRS tumors (Figure S5G), suggesting that CAF‐rich microenvironments may facilitate or coexist with genomic instability. Collectively, these findings establish CRS as a comprehensive biomarker reflecting stromal–immune–genomic interactions in GC.

## 4. Discussion

Our study establishes CTHRC1+ CAFs as a critical stromal determinant of immune exclusion and immunotherapy resistance in GC. Through integrative analysis of multicohort scRNA‐seq, spatial transcriptomics, and bulk transcriptomic data, we identified eight transcriptionally distinct CAF subpopulations, with CTHRC1+ CAFs exhibiting selective tumor enrichment, pronounced myofibroblastic features, and the strongest association with T cell exclusion.

Our classification of CAF subpopulations aligns with established myCAF, iCAF, apCAF, and vCAF paradigms while revealing context‐specific specializations. Notably, CTHRC1+ CAFs demonstrated the most significant tumor enrichment and strongest correlation with immune exclusion compared with other myCAF‐like subsets, highlighting the importance of marker‐defined subpopulations for precise therapeutic targeting. Zhang et al. [29] reported that CTHRC1+ CAFs in lung cancer promote drug resistance via a CTHRC1/glycolysis/H3K18la positive feedback loop, and Lu et al. [30] reported that CTHRC1+ fibroblasts promote EMT through WNT5A signaling in colorectal cancer. These findings collectively highlight the pivotal role of CTHRC1+ CAFs as a conserved, functionally aggressive stromal component across cancer types, whose presence may serve as a biomarker for stromal activation and immune evasion.

In our study, we performed pseudotemporal and gene regulatory network analyses to provide mechanistic insights into this process. The bifurcated trajectory from LUM+ CAF progenitors toward CTHRC1+ CAF or MIF+ CAF terminal states reveals distinct functional commitments to ECM remodeling versus inflammatory signaling. The identification of BHLHE41 as the most specifically activated transcription factor in CTHRC1+ CAFs implicates this factor as a master regulator of myofibroblastic activation, consistent with its known roles in hypoxia response and metabolic reprogramming. This connection is biologically coherent, as BHLHE41 is known to drive hypoxia adaptation and glycolytic switching—processes prominently activated in CTHRC1+ CAFs—and to promote myofibroblastic differentiation, consistent with their robust ECM remodeling and TGF‐*β* signatures.

The spatial organization of CTHRC1+ CAFs at the tumor–stroma interface exemplifies stromal barriers in immune‐excluded tumors. These fibroblasts form dense niches that physically segregate immune cells from malignant epithelium, positioning them as critical gatekeepers of immune accessibility. Mechanistically, our analyses identify that MIF signaling may serve as the predominant pathway through which CTHRC1+ CAFs mediate immune suppression. The high expression of MIF by CTHRC1+ CAFs, alongside its receptors on T/NK cells and myeloid cells, establishes a paracrine axis that may directly inhibit T cell function while promoting immunosuppressive myeloid polarization.

The clinical translation of our findings is supported by the CRS, which robustly predicts immunotherapy response and survival across independent cohorts. The stark contrast in response rates between low‐CRS and high‐CRS patients shows CRS as a powerful stratification tool. Integration with TMB refined prognostic discrimination and reflects the multifactorial nature of immunotherapy resistance. Drug sensitivity profiling reveals that high‐CRS tumors exhibit enhanced sensitivity to dasatinib and other compounds targeting CAF‐activated pathways, supporting a precision medicine approach combining stroma‐targeted agents with ICB.

Beyond patient stratification, our findings nominate CTHRC1+ CAFs and the MIF axis as direct therapeutic targets. Pharmacological inhibition of MIF or its receptors (e.g., CXCR4) could disrupt CAF‐mediated immune suppression, while targeting the master transcription factor BHLHE41 may reverse the myofibroblastic phenotype. However, key challenges include CAF heterogeneity (potential compensatory upregulation of other subsets), pathway redundancy, on‐target toxicity in normal tissues, and the need for CAF‐selective delivery strategies. Future preclinical studies using patient‐derived models are warranted to evaluate the efficacy and safety of combining MIF/BHLHE41 blockade with immunotherapy. Although no agent directly targets CTHRC1, several existing drugs can disrupt the CTHRC1+ CAF‐MIF axis. MIF inhibitors (ISO‐1, 4‐IPP) and CXCR4 antagonists (plerixafor) have shown preclinical activity against CAF‐mediated immune exclusion. Dasatinib, identified by our drug sensitivity analysis, is a clinically available kinase inhibitor that depletes CAFs and enhances ICI efficacy. Regarding clinical trials, CXCR4 antagonists (NCT04181827) and TGF‐*β* inhibitors (NCT01582269) are being tested in combination with ICIs in GC, but no trial has yet specifically selected patients based on CTHRC1+ CAF abundance. Our CRS biomarker thus fills this gap by enabling prospective stratification for future stroma‐targeted combination trials.

Limitations include reliance on transcriptional associations without functional validation, and the specific contribution of MIF signaling requires experimental confirmation. To address these gaps, we propose a series of future experimental validations. First, cell coculture assays will be performed by coculturing primary CTHRC1+ CAFs (or CAFs with BHLHE41 knockdown) with autologous T/NK cells to directly assess suppression of T cell proliferation, activation, and cytotoxicity. Second, patient‐derived GC organoids cocultured with CTHRC1+ CAFs will be used to evaluate targeted therapy responses, including MIF inhibitors, CXCR4 antagonist, and dasatinib, alone or in combination with ICB treatment. Third, in vivo models such as immunocompetent mouse GC models will be employed to validate the CAF‐MIF axis as a therapeutic target. These future studies will transform our correlative findings into causal mechanistic insights and preclinical proof‐of‐concept for clinical translation.

## 5. Conclusion

In conclusion, this study defines CTHRC1+ CAFs as central orchestrators of immune exclusion through BHLHE41‐driven differentiation and MIF‐mediated immunosuppression. The CRS biomarker enables identification of patients who may benefit from stroma‐targeted combination therapies, offering a promising strategy to overcome immunotherapy resistance in GC.

## Author Contributions

Y.W., L.T., Y.L., H.Y., and C.H.: conception and design; Y.W., L.T., and P.L.: data analysis and interpretation; P.L. and J.X.: writing—original draft; C.Z., C.H., Y.L., and H.Y.: writing—review and editing; C.H., Y.L., and H.Y.: supervision. Y.W. and L.T. have contributed to the work equally and should be regarded as co‐first authors.

## Funding

This study was supported by the Fujian Provincial Medical “Double High” Construction Fund (Min Wei Yi Zheng [2021] No. 76).

## Disclosure

All authors consent to the publication of this study.

## Ethics Statement

This study is based on public datasets, and the ethical approval and consent are not required.

## Conflicts of Interest

The authors declare no conflicts of interest.

## Supporting information


**Supporting Information** Additional supporting information can be found online in the Supporting Information section. Supporting Information figures and legends. This file contains Figures S1–S5 and their corresponding legends, including quality control and basic characterization of the integrated single‐cell RNA‐seq atlas, extended characterization of CAF heterogeneity and functional states, pseudotemporal trajectory and transcriptional regulatory analyses of CAF subtypes, additional spatial and molecular analyses of CTHRC1+ CAFs, and CRS‐associated stromal, immune, and genomic features.

## Data Availability

The data that support the findings of this study are available from the corresponding authors upon reasonable request.
