# Supplementary material for: Multiomics Analysis Reveals CTHRC1+ CAFs Drive Immunosuppressive Niches and Predict Immunotherapy Resistance in Gastric Cancer
Source: Hum Mutat. 2026 Jul 18;2026:2370955. doi: 10.1155/humu/2370955 (PMC13379890; doi:10.1155/humu/2370955)
Supplement: Supplementary file 1 — Supporting Information Additional supporting information can be found online in the Supporting Information section. Supporting Information figures and legends. This file contains Figures S1–S5 and their corresponding legends, including quality control and basic characterization of the integrated single‐cell RNA‐seq atlas, extended characterization of CAF heterogeneity and functional states, pseudotemporal trajectory and transcriptional regulatory analyses of CAF subtypes, additional spatial and molecular analyses of CTHRC1+ CAFs, and CRS‐associated stromal, immune, and genomic features. [file HUMU-2026-2370955-s001.docx]

**Supplementary Figures and Legends**


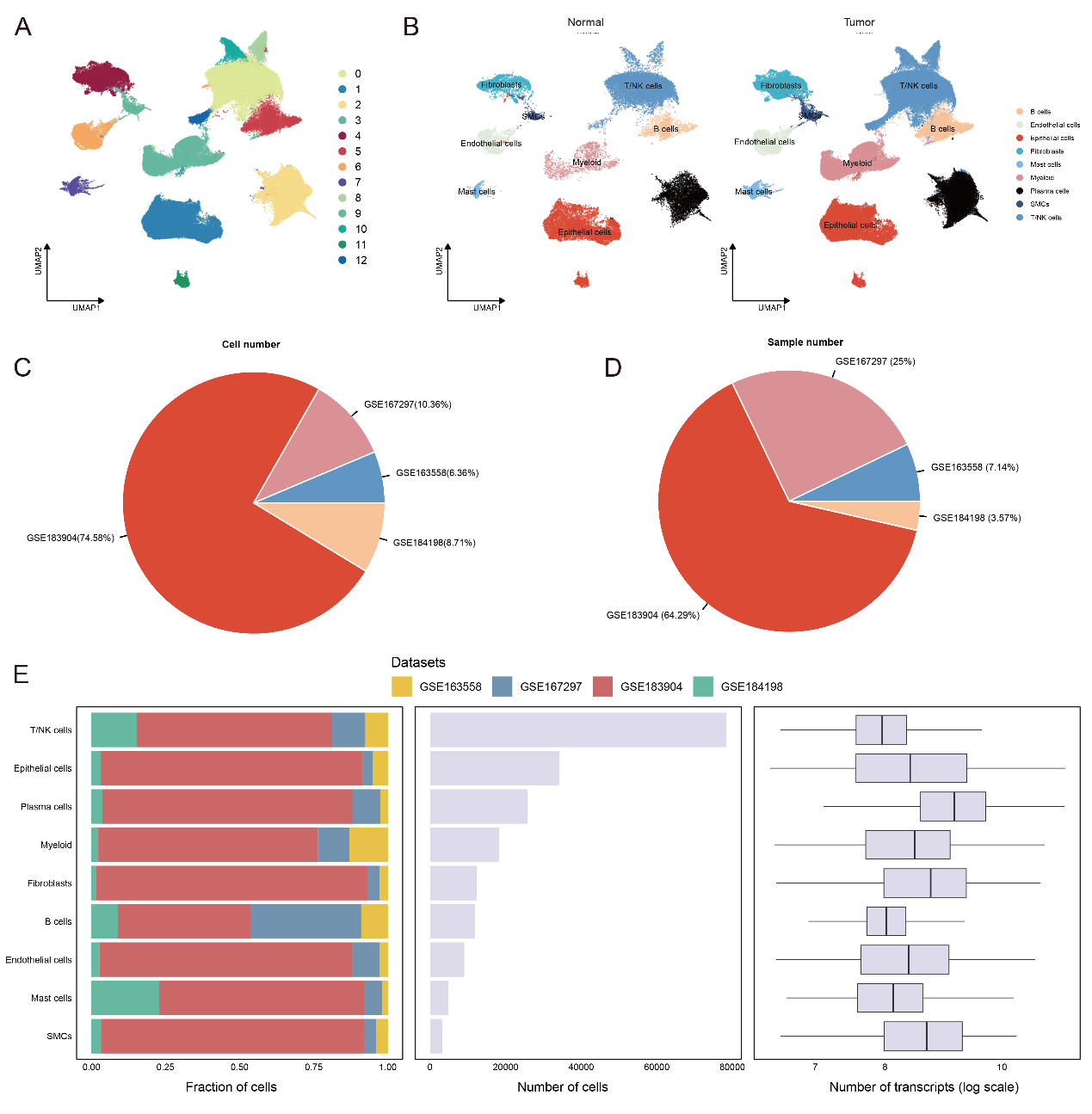


**Figure S1. Quality control and basic characterization of the integrated single-cell RNA-seq atlas for gastric cancer.**
**(A)** UMAP plot shows 13 initial cell clusters are identified from the integrated dataset of 198,199 cells across four cohorts (GSE163558, GSE167297, GSE183904, GSE184198).
**(B)** UMAP plot of the same cells annotated into nine major cell types in the tumor sample and normal sample.
**(C)** Pie chart shows the proportion of total cells contributed by each source dataset.
**(D)** Pie chart shows the proportional contribution of each dataset to the nine major cell types.
**(E)** Bar plots show the distribution of cell counts per sample across datasets. (Right) Box plots show the distribution of unique molecular identifier (UMI) counts per cell across the nine major cell types, indicating consistent sequencing depth.


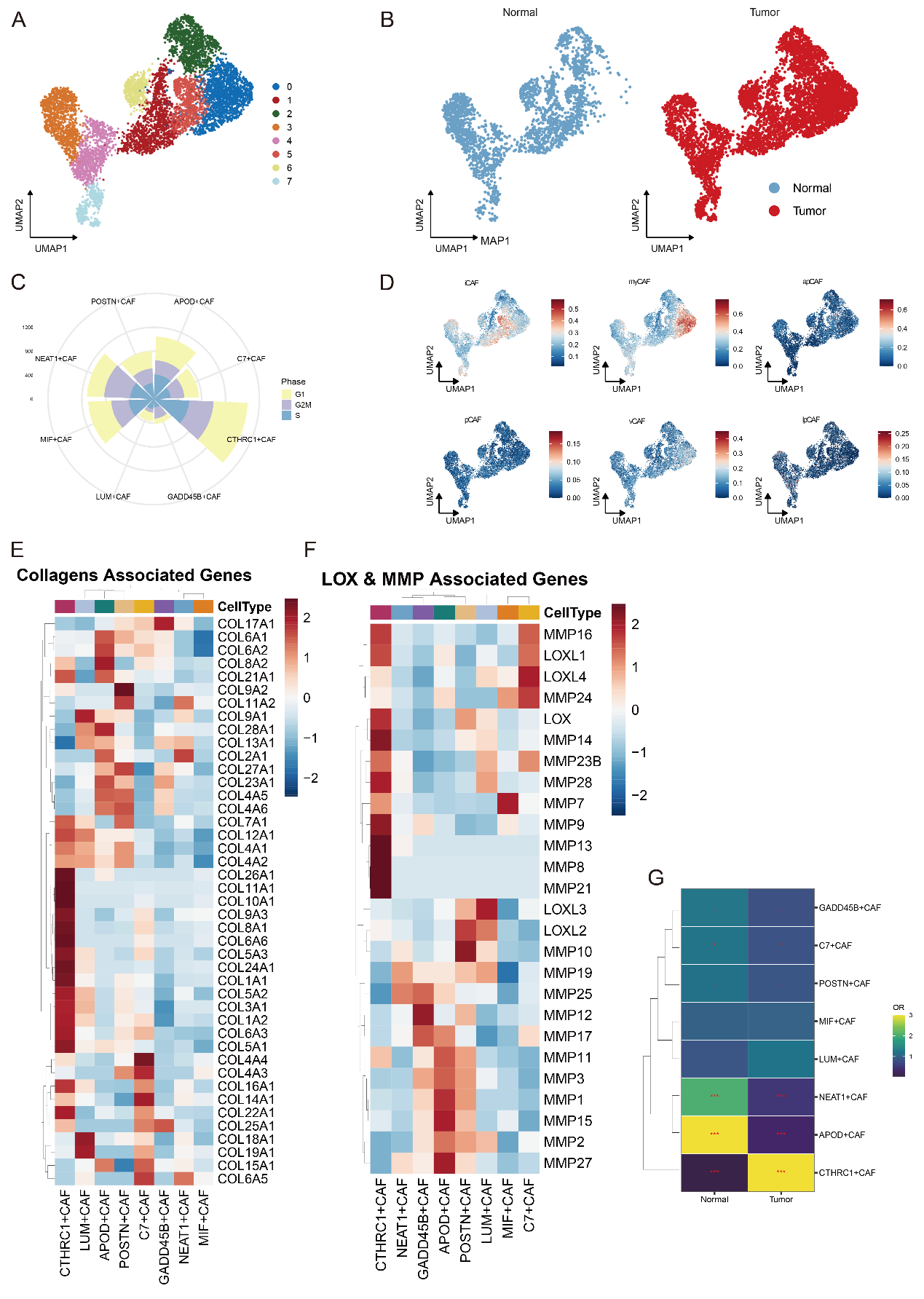


**Figure S2. Extended characterization of CAF heterogeneity and functional states.**
**(A)** UMAP plot of re-clustered fibroblasts, showing eight CAF subtypes.
**(B)** UMAP plots split by tissue origin (Normal vs. Tumor), visually demonstrating the selective enrichment of specific CAF subtypes in tumor tissues.
**(C)** Radar chart shows the proportion of cells in different cell cycle phases (G1, S, G2M) for each CAF subtype. CTHRC1+ and POSTN+ CAFs show higher proliferative fractions.
**(D)** UMAP plots depicts the AUCell enrichment scores of established CAF signatures (myCAF, iCAF, apCAF, vCAF) across the eight subtypes.
**(E)** The expression levels of key extracellular matrix (ECM) genes (such as *COL1A1 and* *COL3A1*) across CAF subtypes.
**(F)** The expression levels of matrix metalloproteinase genes (such as *MMP11 and MMP14*) across CAF subtypes.
**(G)** A heatmap shows the odds ratio (OR) scores that quantify the enrichment of each CAF subtype in tumor versus normal tissues.


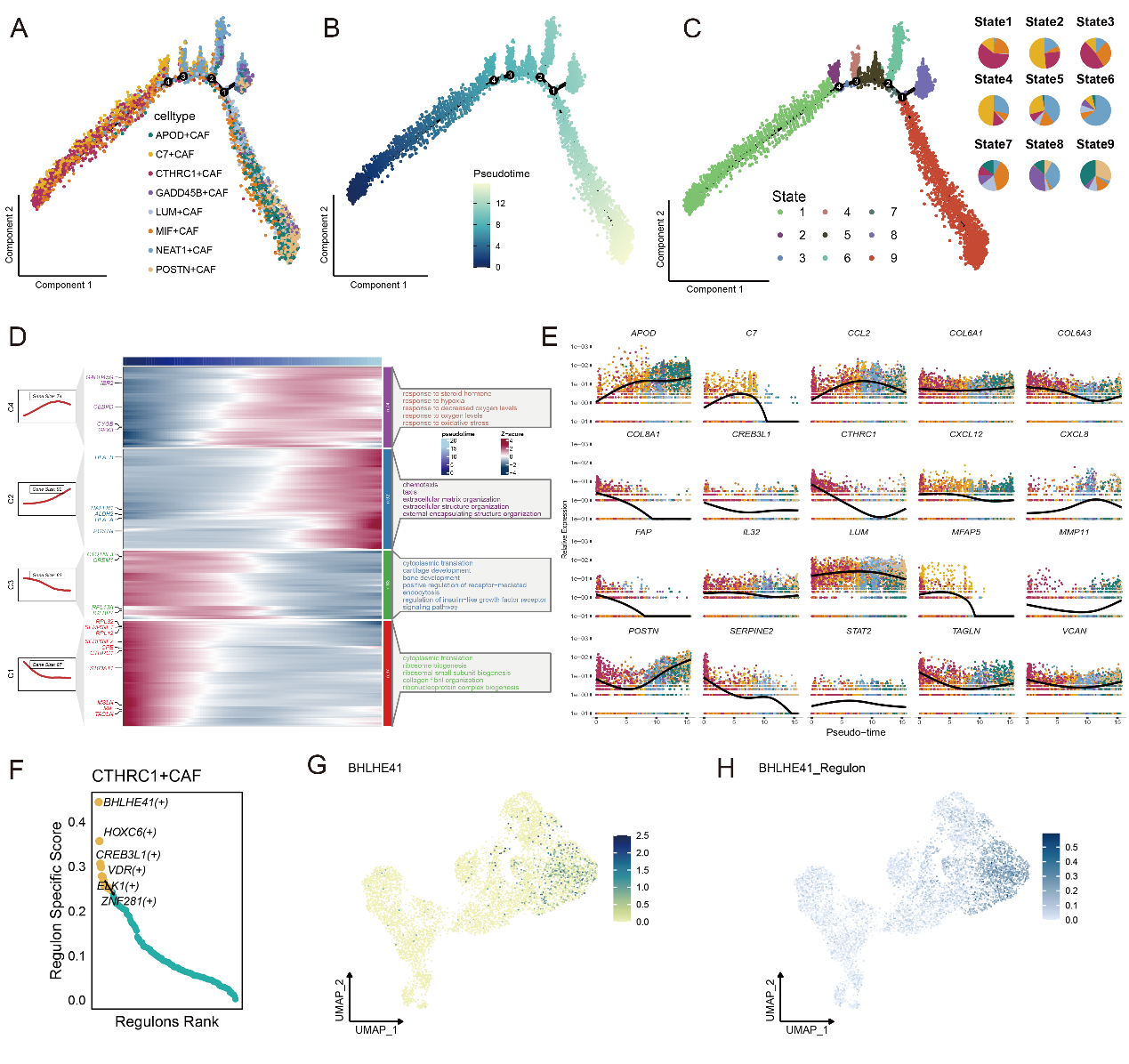


**Figure S3. Developmental trajectory and transcriptional regulation of CAF heterogeneity.**

**(A)** Pseudotemporal trajectory analysis of CAFs shows a bifurcated differentiation path from LUM+ CAF towards CTHRC1+/POSTN+ CAF and MIF+ CAF branches. Cells are colored by subtype.

**(B)** Same trajectory with cells colored by pseudotime (from dark to light).

**(C)** CAF subtypes across nine distinct cellular states defined along the trajectory.

**(D)** (Left) Heatmap of gene expression dynamics along pseudotime, clustered into four major patterns (Clusters 1-4). (Right) GO biological process terms enriched in early versus late pseudotime states.

**(E)** Expression patterns of representative genes from Clusters 1 (LUM, DCN) and 4 (POSTN, VEGFA) along pseudotime and across trajectory branches.

**(F)** Bar plot of regulon specificity scores (RSS) identifies the top master transcription factors for CTHRC1+ CAF subtype.

**(G)** UMAP plot highlights the specific expression of BHLHE41 in the CTHRC1+ CAF cluster.

**(H)** UMAP plot highlights the regulon score of BHLHE41 in the CTHRC1+ CAF cluster.


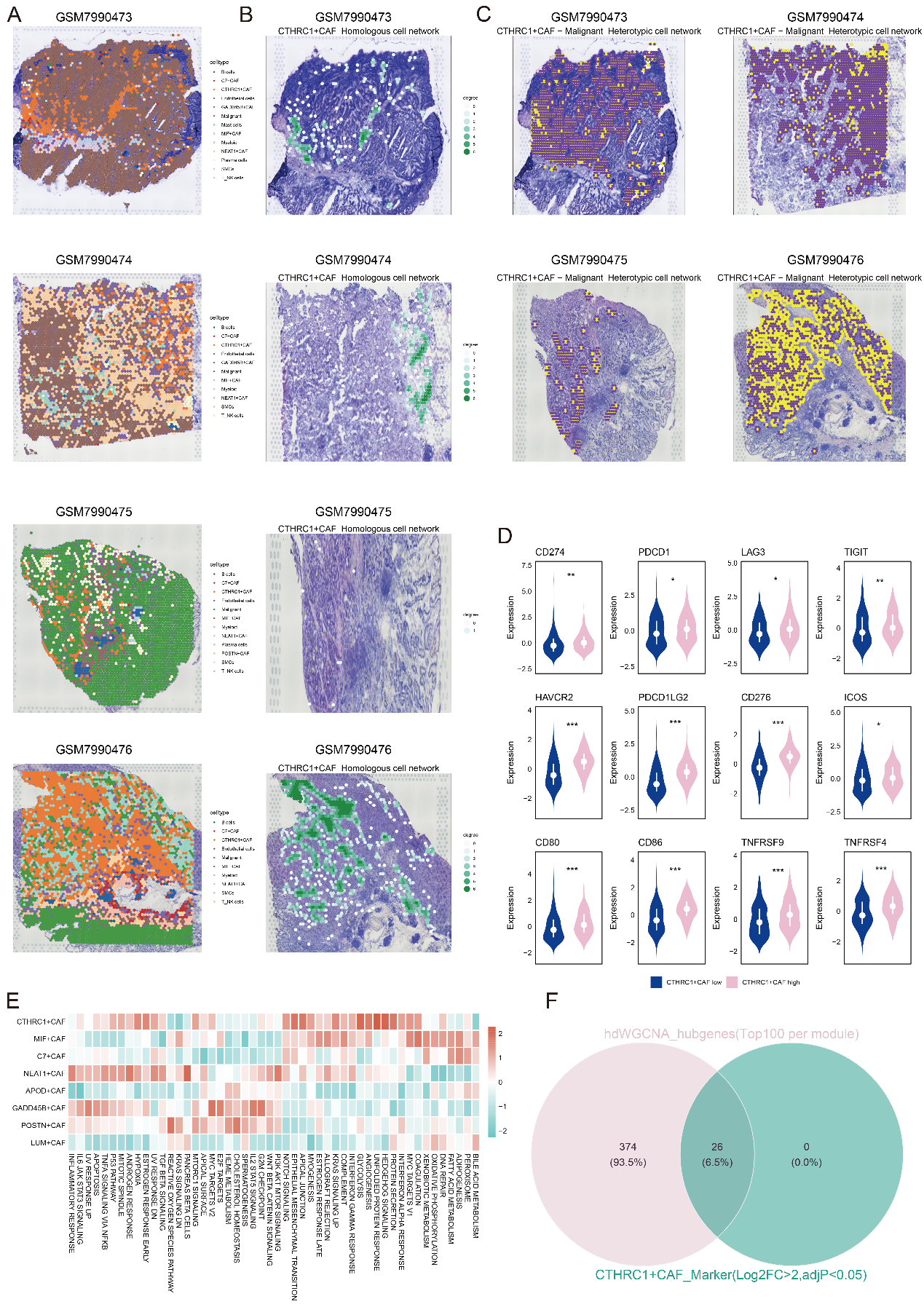


**Figure S4. Additional spatial, molecular, and signature-related analyses.**
**(A)** Spatial distribution maps of major cell types (Malignant cells, Fibroblasts, T/NK cells, Myeloid cells) across four gastric cancer spatial transcriptomics samples (GSM7990473-GSM7990476), as deconvoluted by RCTD.
**(B)** Spatial feature plot highlights the distribution pattern of CTHRC1+ CAFs in a representative sample.
**(C)** Homologous cell network analysis demonstrates the spatial clustering and interaction patterns of CTHRC1+ CAFs.
**(D)** Violin plots compares the expression levels of multiple immune checkpoint molecules (e.g., CD274/PD-L1, PDCD1/PD-1, *LAG3*, *TIGIT*) between CTHRC1+ CAF-high and CTHRC1+ CAF-low tumors in the TCGA-STAD cohort.
**(E)** Heatmap of Gene Set Variation Analysis (GSVA) scores for Hallmark pathways across the eight CAF subtypes.
**(F)** Venn diagram illustrates the overlap between genes from hdWGCNA modules and CTHRC1+ CAF marker genes, which formed the basis for constructing the CTHRC1+ CAF-Related Risk Signature (CRS).


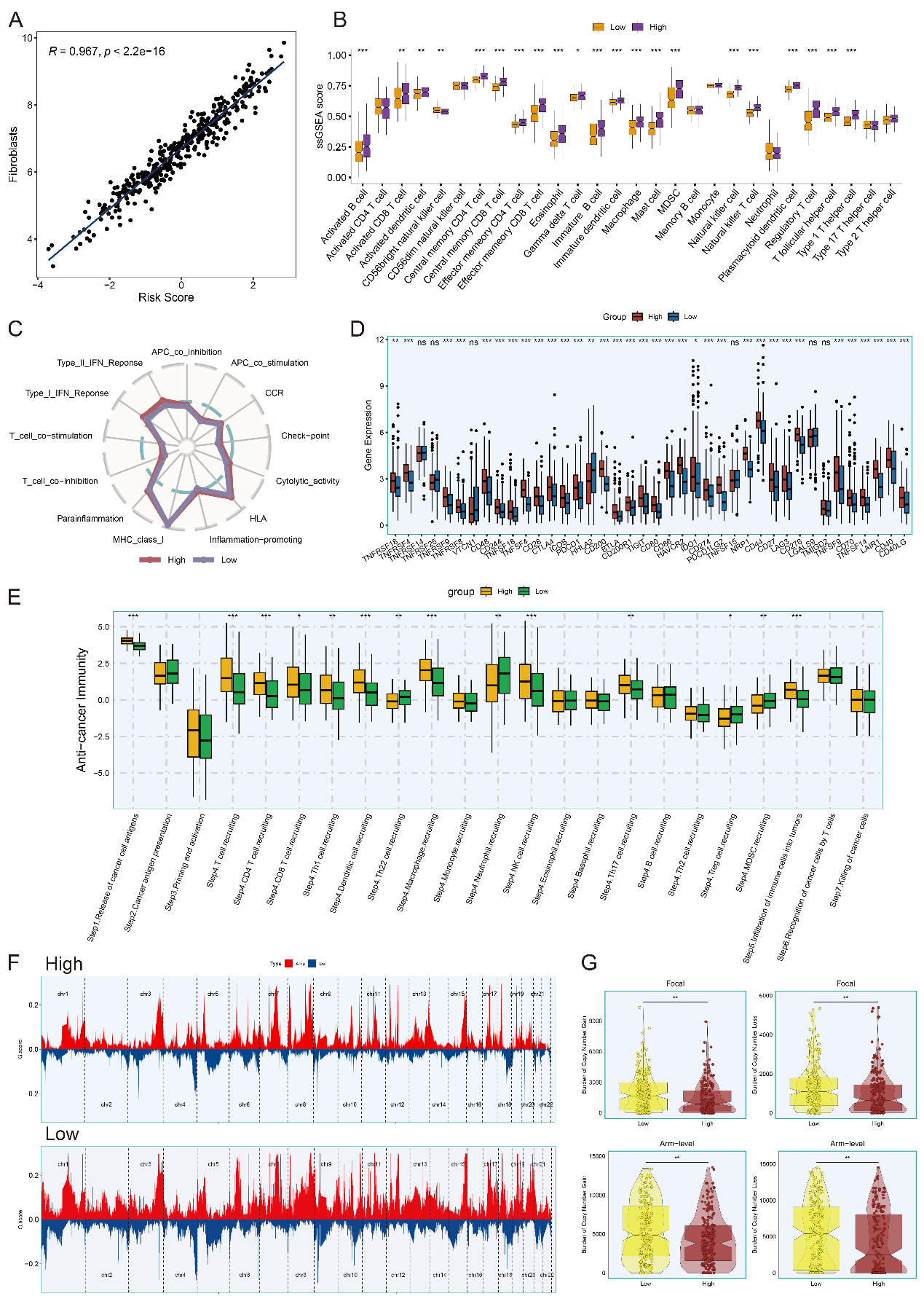


**Figure S5. Comprehensive analysis of the CRS and associated stromal-immune-genomic features.**
**(A)** Scatter plot shows a strong positive correlation (Pearson R=0.967) between the CRS and fibroblast abundance scores derived from MCPcounter in the TCGA-STAD cohort.
**(B)** Box plots compare the infiltration levels (ssGSEA scores) of various immune cell subsets between high- and low-CRS tumors.
**(C)** Radar chart compares the activity scores of key immune-related pathways between high- and low-CRS tumors.
**(D)** Box plots compare the expression levels of additional immune checkpoint molecules between high- and low-CRS groups.
**(E)** Box plots show the activity scores of different steps in the cancer-immunity cycle between high- and low-CRS tumors.
**(F)** Copy number variation (CNV) frequencies (GISTIC2.0 scores) comparing high- vs. low-CRS tumors, highlighting regions of differential amplification (red) and deletion (blue).
**(G)** Box-violin plots compare the rates of focal and arm-level copy number alterations between high- and low-CRS tumors.
